# Daily associations between affect and cognitive performance in older adults with depression and cognitive impairment: a series of seven single-subject studies in the Netherlands

**DOI:** 10.1186/s12877-022-02797-y

**Published:** 2022-02-17

**Authors:** Alieke Tieks, Richard C. Oude Voshaar, Marij Zuidersma

**Affiliations:** 1 University of Groningen, University Medical Center Groningen, Interdisciplinary center Psychopathology and Emotion regulation, Groningen, the Netherlands; 2 Department of Psychiatry, HPC CC72, University of Groningen, University Medical Center Groningen, PO Box 30001, 9700 RB Groningen, Netherlands

**Keywords:** Single-subject, Idiographic, Depression, Cognition

## Abstract

**Background:**

Comorbidity between depression and cognitive impairment is common in older adults, increases the disease burden disproportionally, and leads to diagnostic uncertainty. Insight into individual daily associations between affect and cognitive performance may help in personalizing diagnosis and treatment decisions. Our objective was to get insight into the daily associations between affect and cognitive performance within individual older adults.

**Methods:**

In this single-subject study seven older adults with both depression and cognitive impairment filled in electronic diaries daily for 62-93 consecutive days evaluating positive affect (PA), negative affect (NA), working memory (WM) and visual learning (VL). Time-series analyses using vector autoregressive modelling, Granger causality tests and cumulative orthogonalized impulse response function analyses were performed for each individual separately.

**Results:**

In one patient higher NA was associated with better WM the next day. For another patient days with higher NA and lower PA were days with worse WM. For a third patient better VL was associated with lower NA and higher PA the next day. No associations were found for four patients.

**Conclusions:**

These results highlight heterogeneity in the daily associations between affect and cognitive performance and stress the relevance of single-subject studies. These studies may be an important step towards personalized diagnosis and treatment in old age psychiatry.

**Supplementary Information:**

The online version contains supplementary material available at 10.1186/s12877-022-02797-y.

## Introduction

Patients with both depression and cognitive impairment form a vulnerable patient group, as the combination of depression and cognitive impairment has been associated with a poorer response to antidepressant medication [1, 2], an increased risk of functional dependence [3], and an increased risk of subsequently developing dementia [4, 5]. While cognitive impairment is generally seen as a first manifestation of an underlying cognitive disorder [6], cognitive impairment in the context of depression may also reflect a psychological reaction to cognitive decline [7, 8] or a contemporaneous consequence of the depressive state (i.e. state hypothesis and pseudodementia) [9–11]. To get insight into the nature of the association between depression and cognitive impairment for individual patients, it may be relevant to study bi-directionally the associations between affect and cognitive performance within patients on a daily basis.

Affect has been found to be associated with cognitive performance on a daily basis in several ways. Both negative affect (NA) and positive affect (PA) were associated with worse cognitive performance [12, 13], which may be explained by a reduction of attentional resources needed for execution of cognitive tasks [14]. However, beneficial effects of PA on cognitive performance have also been reported, which may be explained by an increase in motivation [15]. Affect may also influence cognitive performance the next day, as poor mood states may induce negative next-day effects on cognition, for example through a low level of physical activity [16] or a disturbed sleep [17, 18]. Collectively, these findings suggest that cognitive impairment is a consequence of the affective state. In these cases, treatment focused on affect may improve depression as well as cognition. In addition, experiencing a decline in one’s cognitive performance or being confronted with cognitive limitations can evoke worse affective states which may persist the next day(s). In these circumstances, treatment could be focused on acceptance and adjustment to the cognitive impairments [19].

Finally, it is likely that individual differences exist in the temporal order of these associations. Previous studies have largely ignored this potential heterogeneity by using a nomothetic approach in which group averages are calculated and results of all individuals are aggregated. In the presence of individual differences, results of studies using aggregated results will not apply to individual patients [20, 21]. To obtain results that apply to an individual patient, the individual should be the unit of analysis [20]. In a single-subject study analyses are performed for each individual separately and intensive longitudinal data are collected. As a consequence, single-subject designs are able to capture the highly dynamic nature of variables of interest on a within-person level, and yield results that apply to individual persons [20, 22]. If repeated in a sufficient number of participants, results from single-subject studies may be generalizable to the population [22].

The objective of the current study was to get insight into the individual daily associations between affect and cognitive performance in older adults using a single-subject design. We hypothesized that the direction, sign and size of the associations between affect and cognitive performance differ per person.

## Methods

### Study design

We used a single-subject time-series design measuring affect and cognitive performance on a daily basis among patients with cognitive impairment and depressive symptoms who had participated in the idiographic study on Cognitive function, Affect and Sleep in the Elderly (iCASE) [23] (METC 2013/019), which aimed to identify temporal associations between sleep, depression and cognitive performance. In accordance with the single-subject study design, each patient was measured over time intensively and time series data of each patient were analyzed separately. Patients filled in electronic diaries in their home environment for 63 consecutive days including a questionnaire evaluating positive affect, negative affect, and a brief cognitive test battery. We obtained written informed consent from each patient and the study protocol was approved by the institutional review board of the University Medical Center Groningen (UMCG).

### Study population and recruitment

Patients were recruited from the memory clinic and psychiatry department of the UMCG and Lentis, a specialized mental health care institute in Groningen. Inclusion criteria were 1) ≥60 years old, 2) major depressive disorder according to the DSM-IV criteria or a score of ≥4 on the Geriatric Depression Scale (GDS), and 3) mild cognitive impairment (MCI) or mild dementia diagnosed by the multidisciplinary team of a memory clinic, or a Mini Mental State Examination (MMSE) [24] score of <25, or a Montreal Cognitive Assessment (MOCA) [25] score of <26. Patients were excluded if they had a severe medical illness influencing short-term survival; a history of bipolar or psychotic disorders, and/or substance use disorder in the past two years; a clinical dementia rating scale of >1; were unable to participate (e.g. due to language impairments); were mentally incompetent to give consent for study participation.

### Procedures

The baseline assessment was conducted once at the start of the study at the patient’s home. During this assessment, several questionnaires and interviews (including the GDS and MOCA) were administered and the patients received instructions for filling out the diaries [23]. During the 63-day study period, patients filled out an electronic diary on a laptop once every evening one hour before going to bed. The daily diary consisted of two components: 1) questionnaires about affect, depressive symptoms (PHQ-8) [26], behavioral and contextual variables, and 2) an online brief cognitive test battery which was accessed via a hyperlink at the end of the diary-questionnaires*.* Patients were instructed to fill out the diaries in a quiet area with minimal distraction. The diary took about 20 minutes to complete. All patients were allowed to continue their treatment as usual. A trained research assistant had weekly phone calls with each of the patients to ask whether any important events occurred or whether there were any changes in treatment. The researcher was available by telephone seven days a week in case of questions or problems.

### Measurements

#### Cognitive performance

Cognitive performance was assessed using the Cogstate Brief Battery [27], a computerized neurocognitive test battery, which has been developed specifically for repeated testing of individuals in the absence of supervision and has been validated in older adults with MCI [27]. The Cogstate Brief Battery validly measures a range of cognitive domains while minimizing practice effects [28]. For the current study we included working memory and visual learning.

#### Working memory

We included working memory as this domain fluctuates over time within individuals and has been used in previous studies assessing the daily associations between affect and cognitive performance [12, 13, 29–31]. Working memory was assessed using the One Back Task. In this task a playing card is shown face-down on the screen. As soon as the card is flipped, the patient has to indicate as fast as possible whether the card shown was the same as the previous card (for a video see: https://www.cogstate.com/academic-research/ last accessed: 3 Sep 2021). Reaction time defined as the mean time spent on correct responses was used as the outcome of the task. Reaction times were transformed on a base 10 logarithmic scale to approximate a normal distribution. The inverse was taken so that higher scores represent faster reaction times.

#### Visual learning

Visual learning is the most complex task included in the Cogstate Brief Battery and was chosen since especially cognitively demanding tasks may interfere with affect [14]. Visual learning was assessed using the One Card Learning Task. In this task a playing card is shown face-up on the screen. As soon as the card is flipped, the patient has to indicate as fast as possible whether they have seen this card before in the task (for a video see: https://www.cogstate.com/academic-research/ last accessed: 3 Sep 2021). Accuracy of performance, defined as the proportion of correct responses, was used as the outcome of the task. Proportions were transformed on an arcsine scale to approximate a normal distribution.

#### Affect

We assessed momentary self-reported positive- and negative affect. Affect was measured by six positive affect items (I feel relaxed; energetic; enthusiastic; content; calm; cheerful), and six negative affect items (I feel gloomy; anxious; nervous; irritable; dull; tired) [32]. Items were measured on a Visual Analogue Scale ranging from 0 (“not at all”) to 100 (“very much”). Sum-scores for positive- and negative affect were calculated by summing the scores of the separate items creating a score with a possible range of 0 to 600.

#### Potential confounders

We included a change in psychiatric treatment as a potential confounder since this might influence both affect and cognitive performance [11, 33]. Psychotropic drug use was explored during the weekly phone calls and changes (switch or dosage) were verified by the treating specialist. A change in medication dosage was included as a continuous time-series variable. A change in starting/stopping of treatment or starting/ending admittance was included as a dichotomous time-series variable (0 = no treatment, 1 = treatment).

### Statistics

#### VAR models

To get insight into the individual daily associations between affect and cognitive performance, we performed time-series analyses using Vector Autoregressive (VAR) modelling for each patient separately. VAR models are multivariate autoregressive models consisting of a set of regression equations and are particularly suitable to study the temporal dynamics of a system of one or more variables. Using such models, the dynamic part of the model (i.e., next-day effects) can be separated from the simultaneous part (i.e., associations on the same day) [34]. Separate VAR models were constructed for negative affect (NA) with working memory (WM), positive affect (PA) with WM, NA with visual learning (VL), and PA with VL. In this way four VAR models were constructed for each patient. NA, PA, WM and VL were modelled as endogenous variables, meaning they can be both predictor and outcome. This is important since we do not yet know the direction of the association (i.e., changes in affect might precede changes in cognitive performance, and vice versa). If there were changes in a patient’s treatment, we included this as an exogenous variable (i.e., predictor only) in all models for that patient.

**Fig. 1:**
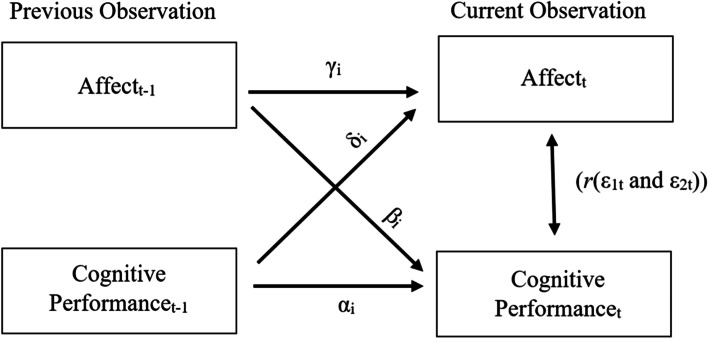
Visualization of the VAR Models for Affect and Cognitive Performance Constructed for Each Patient. *Note.* Affect was either negative- or positive affect. Cognitive performance was either working memory or visual learning. α_i_ and γ_i_ represent autocorrelations. β_i_ and δ_i_ represent cross-lagged associations. *r* represents the contemporaneous associations as the correlation between the residuals ε

Each of the endogenous variables were regressed on their own lagged (i.e., previous) values, which represents the autocorrelations (α_i_ and γ_i_ in Fig. 1); the lagged values of the other endogenous variable, which represents the cross-lagged associations (β_i_ and δ_i_); and on the contemporaneous (i.e., simultaneous) and lagged values of the exogenous variables. The correlation between the residuals (ε_1t_ and ε_2t_) can be interpreted as the contemporaneous associations between the endogenous variables, as the residuals should be serially uncorrelated but can be contemporaneously correlated [34].

The number of lags included in the models was one, which is equivalent to one day, as we expected the values of affect and cognitive performance of the same or the previous day to be most relevant for the current values of affect and cognitive performance. In a single-subject design the statistical power depends on the number of repeated measurements, instead of the number of participants. However, the expected effect size and even its direction are generally unknown in time-series analyses [34]. Simulation studies have shown that 30 repeated measurements are sufficient for time-series analysis [35], while a number of 50 repeated measurements is often used as a rule of thumb [36]. We have chosen a 9 week (63 days) study period to take into account potential missing data.

#### Model selection

The VAR models were constructed using the ‘autovar’ R-package [37, 38]. This package tests all possible models within given restrictions defined by the researcher, checks assumptions and summarizes outcomes of all models that meet the four assumptions (i.e., valid models) (see Appendix).

#### Statistical analyses

Estimates of VAR models are part of a dynamical system and cannot be interpreted separately [34]. Therefore, we performed Granger causality tests and Cumulative Orthogonalized Impulse Response Function (COIRF) analyses [34, 35, 39]. The Granger causality test was performed to assess the direction of the associations between affect and cognitive performance. This test determines whether previous values of X predict current values of Y better than previous values of Y alone. When this is the case, X is said to ‘Granger cause’ Y [35, 39]. The sign of the associations (i.e., positive or negative) was assessed using the estimates from the VAR models. COIRF analyses were performed to determine and visualize the strength of the associations. This analysis visualizes the cumulative effects of an impulse or ‘shock’ of one standard deviation increase in one of the endogenous variables on the other endogenous variables in the model over a certain period [34]. We examined the effects over a 10-day period. The analysis makes an assumption about the specific order of the contemporaneous associations which has to be specified. Because affect and cognitive performance were assessed right after one another and the direction of their contemporaneous associations were unknown, we examined both orders. COIRF analyses were performed only for patients with significant Granger causality.

Statistical significance was defined as *p*<.01 to correct for multiple testing. Analyses were performed using R studio version 1.2.5019. For more detailed information about data preparation and model selection, we refer to the Appendix.

## Results

### Sample characteristics

During the inclusion period between January 2015 and June 2019, 96 eligible patients were asked to participate. Of these, 83 did not want to participate or did not respond to the invitation letter, and 13 started the study (13.5%). Of these 13, one stopped after a few days, so the initial sample was 12 participants who completed the main study. Of these twelve, three were excluded from the present study because they had no data about affect (participants 1, 2 and 3), one (participant 6) refused to perform the cognitive tests, and one (participant 12) was excluded because, in hindsight, this participant did not meet criteria for cognitive impairment. The final sample used for the current study consisted of seven patients (participants 4, 5, 7, 8, 9, 10 and 11) (three women, four men). The age ranged from 61 to 83 years. The GDS score at baseline ranged from 5 to 12. Patients filled in their diaries for a period of 62 to 93 consecutive days (Table 1).

**Table 1:** Baseline characteristics and endogenous variables of all patients

Patient	4	5	7	8	9	10	11
Baseline Characteristics							
Age	83	62	71	69	70	68	61
Sex	Male	Female	Male	Female	Male	Female	Male
Highest completed Education	General Secondary Education	Lower Vocational Education	University	Higher Professional Education	Lower Vocational Education	Higher Professional Education	Secondary Vocational Education
MOCA^a^/MMSE^b^		24	25	24		30	
GDS^c^	5	11	11	6	10	8	12
Time-series Variables							
Length (days)	93	63	63	62	63	77	70
Working Memory^d^							
Mean (SD) Missings N (%)	744 (98) 3 (3)	638 (96) 2 (3)	648 (7) 2 (3)	618 (5) 12 (19)	639 (31) 14 (22)	912 (84) 11 (14)	721 (181) 23 (33)
Visual							
Learning^e^ Mean (SD) Missings N (%)	0.64 (0.08) 3 (3)	0.72 (0.08) 2 (3)	0.70 (0.08) 2 (3)	0.57 (0.08) 12 (19)	0.80 (0.07) 14 (22)	0.79 (0.08) 11 (14)	0.60 (0.12) 23 (33)
Negative Affect^f^							
Mean (SD) Missings N (%)	216 (28) 1 (1)	370 (31) 2 (3)	418 (52) 2 (3)	101 (91) 6 (10)	72 (28) 3 (5)	75 (54) 11 (14)	303 (55) 18 (26)
Positive Affect^g^							
Mean (SD) Missings N (%)	268 (35) 1 (1)	170 (37) 2 (3)	72 (38) 2 (3)	429 (131) 6 (10)	187 (71) 3 (5)	352 (82) 11 (14)	261 (36) 18 (26)

^a^Montreal cognitive assessment score indicating cognitive performance with a possible range of 0 (worst cognitive performance) to 30 (best cognitive performance). ^b^Mini-mental state examination score indicating cognitive performance with a possible range of 0 (worst cognitive performance) to 30 (best cognitive performance). ^c^Geriatric Depression Scale score indicating severity of depressive symptoms in the past week with a possible range of 0 (no depressive symptoms) to 15 (very severe depressive symptoms). ^d^Working memory reaction time for correct responses on the One Back Task in milliseconds. ^e^Visual learning accuracy indicating the proportion of correct answers on the One Card Learning Task. ^f^Negative affect scores measured as the sum-score of six negative affect items with a possible range of 0 (‘not at all’ on all items) to 600 (‘very much’ on all items). ^g^Positive affect scores measured as the sum-score of six positive affect items with a possible range of 0 (‘not at all’ on all items) to 600 (‘very much’ on all items)

### Working memory (WM)

For five out of seven patients, we found no significant cross-lagged or contemporaneous associations between affect and working memory (Table 2). For patient 4 negative affect (NA) significantly Granger caused WM (*p*=.005), in which higher NA on the current day was associated with better WM the next day (VAR estimate = 0.246, see Table 2). For patient 8 there was a significant contemporaneous association between NA and WM and PA and WM. Days with higher NA were days with worse WM (*r*=-.37; *p*=.003), and days with higher PA were days with better WM (*r*=.35; *p*=.005).

**Table 2:** Granger Causality Tests and Contemporaneous Associations for Working Memory and Affect for All Patients

Patient	Granger Causality VAR Estimate (*p*-value)^a^				Contemporaneous Associations *r* (*p*-value)	
	NA→WM	WM→NA	PA→WM	WM→PA	NA↔WM	PA↔WM
4	**0.246**(.005)	0.010(.94)	0.123(.24)	-0.019 (.83)	-.01 (.95)	.20 (.06)
5	-0.007 (.94)	-0.134 (.37)	0.106 (.32)	0.092 (.50)	.09 (.52)	.09 (.48)
7^b^	-0.070 (.46)	-0.206 (.16)	-0.019 (.79)	0.326 (.15)	.02 (.85)	-.02 (.86)
8^c^	-0.212 (.09)	-0.151 (.27)	0.221 (.12)	0.114 (.32)	**-.37** (.003)	**.35** (.005)
9^d^	-0.224 (.05)	0.289 (.02)	0.097 (.55)	0.108 (.21)	.22 (.08)	.11 (.41)
10	-0.098 (.43)	0.023 (.84)	0.054 (.62)	-0.100 (.37)	-.23 (.05)	.03 (.80)
11^e^	-	-	0.054 (.47)	-0.319(.06)	-	-.08 (.53)

Note. *NA* = Negative affect, *PA* Positive affect, *WM* Working memory. Changes in the variable before the arrow (→) precede changes in the variable after the arrow. Contemporaneous associations are represented by a double-headed arrow (↔)

^a^ *p*-value used to determine statistical significance was derived from the Granger causality test. df = 1 in all Granger causality tests. ^b^PA was transformed on a logistic scale. ^c^NA was transformed on a logistic scale. ^d^NA was transformed on an inverse scale (1/NA). ^e^No valid models constructed due to non-normality of NA. Bold numbers represent statistical significance at *p*<.01

### Visual learning (VL)

For six out of seven patients, we found no significant cross-lagged or contemporaneous associations between affect and visual learning (Table 3). For patient 10 VL significantly Granger caused NA (*p*<.001), in which better VL on the current day was associated with lower NA the next day. VL also significantly Granger caused PA (*p*=.001) in this patient, in which better VL on the current day was associated with higher PA the next day.

**Table 3:** Granger Causality Tests and Contemporaneous Associations for Visual Learning and Affect for All Patients

Patient	Granger Causality VAR Estimate (*p*-value)^a^				Contemporaneous Associations *r* (*p*-value)	
	NA→VL	VL→NA	PA→VL	VL→PA	NA↔VL	PA↔VL
4	-0.097 (.33)	-0.096 (.39)	-0.093 (.43)	0.069 (.34)	-.06 (.56)	.03 (.76)
5	-0.065 (.63)	0.143 (.26)	0.056 (.67)	-0.024 (.82)	.06 (.65)	-.11 (.41)
7^b^	-0.139 (.35)	-0.056 (.58)	0.011 (.89)	-0.000 (.99)	-.18 (.16)	.24 (.06)
8^c^	0.106 (.25)	-0.171 (.30)	0.109 (.40)	0.219 (.04)	-.14 (.29)	.19 (.14)
9^d^	0.016 (.88)	0.035 (.83)	-0.101 (.49)	-0.056 (.57)	-.07 (.61)	-.27 (.03)
10	0.058 (.55)	**-0.506** (<.001)	-0.034 (.69)	**0.450** (.001)	.00 (.98)	-.00 (.98)
11^e^	-	-	-0.114 (.15)	0.051 (.78)	-	.02 (.88)

Note. *NA* negative affect, *PA* positive affect, *VL* Visual learning. Changes in the variable before the arrow (→) precede changes in the variable after the arrow. Contemporaneous associations are represented by a double-headed arrow (↔)

^a^ *p*-value used to determine statistical significance was derived from the Granger causality test. df = 1 in all Granger causality tests. ^b^PA and VL were transformed on a logistic scale. ^c^NA and VL were transformed on a logistic scale. ^d^NA was transformed on an inverse scale (1/NA). ^e^No valid models constructed due to non-normality of NA. Bold numbers represent statistical significance at *p*<.01

### Dynamic effect sizes (COIRF analysis)

#### Working memory

**Fig. 2:**
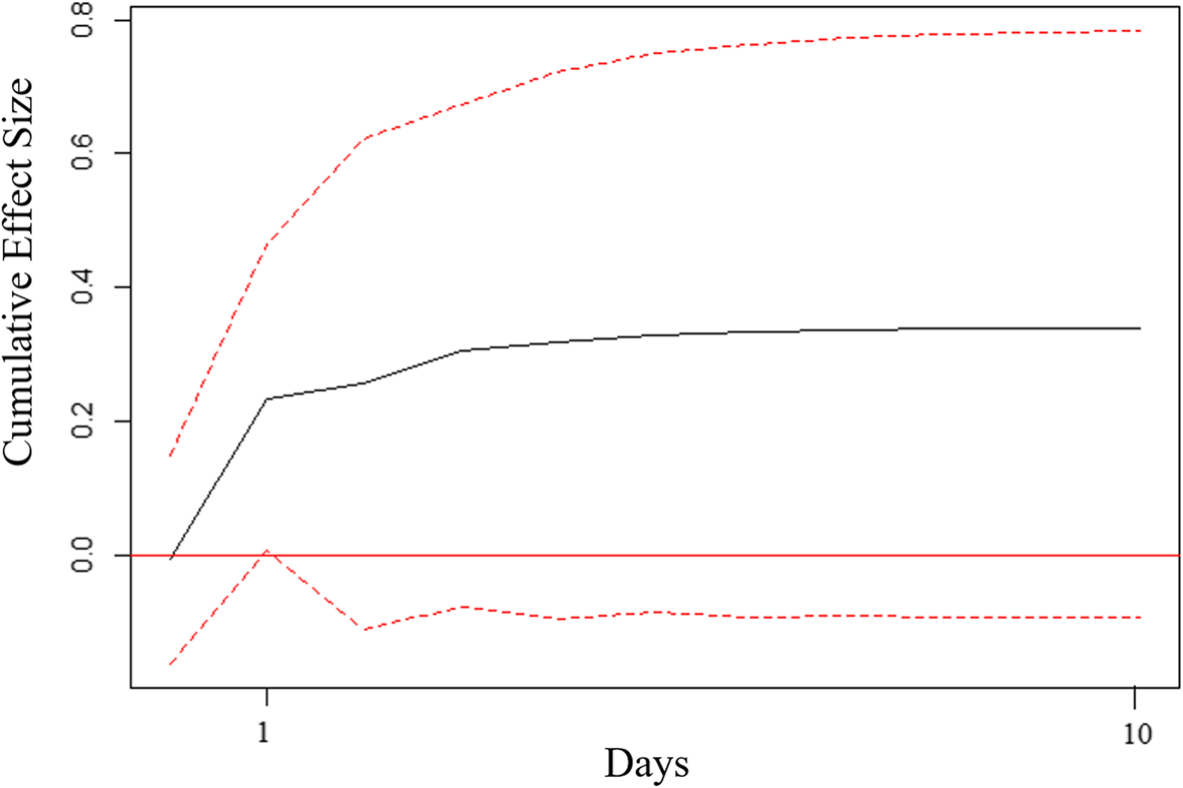
Order 1 Cumulative Orthogonalized Impulse Response Function Analysis of Working Memory and Negative Affect for Patient 4. *Note.* Impulse = negative affect. Response = working memory. Order 1 assumes a contemporaneous association in the direction of changes in negative affect preceding changes in working memory. Dotted lines represent the 99% confidence interval around the cumulative effect sizes

For patient 4 the cumulative effect of an increase of 1 SD in NA resulted in a 0.341 SD (99% CI: -0.092; 0.782) increase in WM over a period of 10 days (Fig. 2). This effect was significant at the *p*<.01 level at the first lag only (after 1 day). Only order 1 results are shown as the ordering of the contemporaneous associations had little effect on the results (see Appendix for order 2 results).

#### Visual learning

**Fig. 3:**
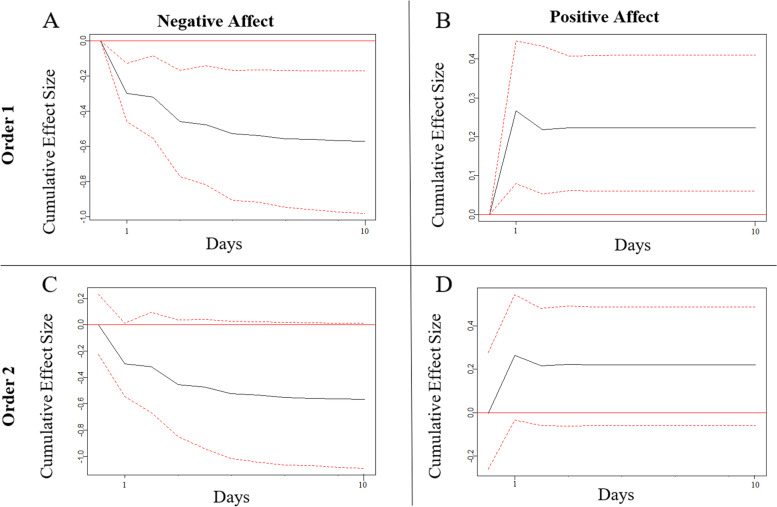
Cumulative Orthogonalized Impulse Response Function Analyses of Visual Learning and Affect for Patient 10, *Note*. In panel A and C: impulse = visual learning; response = negative affect. In panel B and D: impulse = visual learning; response = positive affect. Order 1 assumes a contemporaneous association in the direction of changes in affect preceding changes in visual learning. Order 2 assumes a contemporaneous association in the direction of changes in visual learning preceding changes in affect. Dotted lines represent the 99% confidence intervals around the cumulative effect sizes

For patient 10, assuming order 1 (affect first), the cumulative effect of an increase of 1 SD in VL resulted in a 0.596 SD (99% CI: -0.980; -0.170) decrease in NA and a 0.223 SD (99% CI: 0.060; 0.410) increase in PA over a period of 10 days (Fig. 3). For the order 1 results these effects were significant across all 10 days. For the order 2 results (VL first), none of these effects were significant.

## Discussion

### Main findings

This single-subject study demonstrated that daily associations between affect and cognitive performance can differ between patients with depressive symptoms and cognitive impairment. For three out of seven patients we found significant daily associations between affect and cognitive performance: For one patient we found that an increase in negative affect was associated with better working memory the next day. For another patient we found that days with better working memory were also days with higher positive affect and lower negative affect. For a third patient we found that better visual learning was associated with higher positive affect and lower negative affect the next day. In accordance with our hypothesis, the associations found within these three patients were all different in direction, sign and strength.

### Cross-lagged associations of affect with working memory

For one patient we found that an increase in negative affect was associated with better working memory the next day. This is not in line with theories such as the dual task perspective, which assumes that affect influences cognitive performance by a reduction in cognitive resources [14], or the motivational perspective, which argues that affect influences cognitive performance through motivation [15]. Both of these theories assume an increase in negative affect worsens cognitive performance. However, this finding is in line with results from an earlier study in which an increase in negative affect was associated with better working memory at low levels of negative affect [13]. This association might have an inverted U-shape in which negative affect might be beneficial to cognitive performance only at low levels of affect, for example by temporarily increasing arousal [40]. So, for patients in which an increase in negative affect is associated with better cognitive performance, interventions to reduce negative affect (e.g., relaxation exercises) might be relevant to avoid as long as negative affect states do not interfere with daily functioning.

### Contemporaneous associations of affect with working memory

For another patient we found that days with better working memory were also days with higher positive affect and lower negative affect. These findings seem to fit the motivational perspective, which assumes affect influences cognitive performance through motivation. According to this theory, higher PA is beneficial to cognitive performance through an increase in motivation, while the opposite is true for higher NA [15]. These findings also match some previous findings by Brose and colleagues who showed that days with above average PA and enhanced working memory performance, were also days with above average motivation, arguing for a beneficial effect of PA on working memory performance [29]. So, according to the motivational perspective, we may expect a negative contemporaneous association between NA and cognitive performance, but a positive contemporaneous association between PA and cognitive performance (i.e. at moments when NA is high and PA is low, cognitive performance is worse, and vice versa). Therefore, for patients showing such a contemporaneous association, alleviating depressive symptoms (i.e., improving their affective state) might have a beneficial influence on cognitive performance.

### Cross-lagged associations of affect with visual learning

For another patient we found that better visual learning was associated with higher positive affect and lower negative affect the next day. These findings are in line with the hypothesis that experiencing a decline in one’s cognitive performance may have emotional consequences [7, 8]. This is also in line with results from several previous studies regarding long-term associations between depression and cognitive performance, although this is (according to our knowledge) the first study to evaluate this hypothesis on a daily basis. For example, Van den Kommer and colleagues explored this hypothesis in a longitudinal study following 2299 older adults for 13 years. They found that low levels of information processing speed were indeed associated with an increase in depressive symptoms over time [41]. Several other studies have reported similar results in which cognitive function predicted depressive symptoms in older adults over time, but not the other way around [8, 42, 43]. So, for patients in which a decrease in cognitive performance is associated with a worsening of the affective state, treatment could, instead, be focused on acceptance and developing better coping strategies (e.g., learning memory aids, asking for help) [19].

### No associations

For four out of seven patients we did not find any associations between affect and cognitive performance. For these patients affect and cognitive performance might not influence one another on a daily basis. This is in line with several other studies that did not find any daily associations between affect and cognitive performance at a group level [30, 44–46]. As total scores for affect and cognitive performance showed sufficient variability in all patients (see Appendix), the lack of associations in these patients is likely not the result of a lack of fluctuation and sensitivity. Although affect and cognitive performance were not associated on a daily basis within these patients, they may still be associated over longer time periods (e.g., months or years). That is, cognitive performance may still improve when depression improves over a longer time period [33, 47]. Another possibility is that cognitive impairment is not a direct consequence of the depressive state, but both conditions share the same underlying mechanism like a neurodegenerative disorder or cerebrovascular disease [11, 48, 49]. Therefore, cognitive impairment in these patients should not merely be seen as a manifestation of depression, but should be taken seriously and monitored regularly for further deterioration.

### Methodological considerations

To our knowledge, this was the first study adopting a single-subject study design to assess heterogeneity in the daily associations between affect and cognitive performance for individual patients with depression and cognitive impairment. This enabled us to consider the heterogeneity and fluctuating nature of the associations, which are often expected to be present in psychological phenomena [20, 34]. In addition, this design has high ecological validity providing insight into how these associations exist in the context of patients’ day to day lives, rather than in a lab or simulated context [50]. Furthermore, by selecting patients with depression and cognitive impairment we included a vulnerable group that is often excluded from studies, while it is a group that is often seen as complex in clinical practice. A limitation that is inherent to the single-subject design is that the results cannot be generalized to other patients [51]. However, the aim of this study was not to generalize results to other patients, but to look at associations on a within-person level. Another concern could be the intra-individual validity of measuring instruments. Even if instruments have been validated in group studies, they may not be valid for specific individuals. In the present study, we tried to examine validity by asking participants after the study if they had difficulties with the questionnaires. One patient (participant 7) indicated difficulties with the affect questionnaire. He said to find the questions “confronting, the answer options arbitrary, and difficult to answer exactly how I felt”. In addition, as this concerns an observational study, the temporal associations do not necessarily imply causal associations. Therefore, all clinical interpretations should be interpreted with caution and considered as examples to illustrate how individual data might be used in clinical practice to tailor diagnoses and treatments to individuals. Future randomized controlled studies should test whether diagnostic advices or treatment strategies based on time-series analyses within patients can improve patient outcomes.

## Conclusions and future perspectives

We identified heterogeneity in the direction, strength and sign of the daily associations between affect and cognitive performance within older adults with depression and cognitive impairment. This demonstrates the importance and potential of studying psychiatric phenomena on an individual level. Especially in the field of psychiatry, where most studied phenomena are highly dynamic and heterogeneous, single-subject studies can provide a way to uncover this temporal complexity and highly dynamic nature of our variables of interest on an individual level [22]. Deepening our knowledge of individual patients may help in providing personalized diagnostics and individually tailored treatment advice. For example, observational single-subject studies may yield important information about which factors trigger or maintain certain symptoms at the individual level. Qualitative methods (e.g. interviews) can further deepen this information by providing more context. Furthermore, combining the results of multiple single-subject studies may help link individual-level associations to between-person characteristics. Our study only included 7 patients, which is too small of a sample for identifying commonalities. Therefore, future series of single-subject studies in more participants could evaluate whether different temporal associations between affect and cognitive performance can be related to specific demographic or clinical patient characteristics. Concludingly, with the current trend in technological advancements, applying single-subject designs in clinical practice by monitoring patients (e.g., via smartwatches) daily has become more feasible, and may provide an important first step towards more personalized psychiatric care [20, 22].

## Supplementary Information

**Additional file 1.**

## Data Availability

The data generated and analyzed during the current study are not publicly available to protect patients’ privacy, as patients may be recognized from their intensive longitudinal data. Data are available from the corresponding author on reasonable request.

## Abbreviations

NA: Negative affect
PA: Positive affect
iCASE: Idiographic study of Cognition, Affect and Sleep in the Elderly
UMCG: University Medical Center Groningen
GDS: Geriatric Depression Scale
MCI: Mild cognitive impairment
MMSE: Mini Mental State Examination
MOCA: Montreal Cognitive Assessment
VAR: Vector autoregressive
WM: Working memory
VL: Visual learning
COIRF: Cumulative orthogonalized impulse response function
