# Supplementary material for: Daily associations between affect and cognitive performance in older adults with depression and cognitive impairment: a series of seven single-subject studies in the Netherlands
Source: BMC Geriatr. 2022 Feb 17;22:133. doi: 10.1186/s12877-022-02797-y (PMC8851709; doi:10.1186/s12877-022-02797-y)
Supplement: Supplementary file 1 — Additional file 1. [file 12877_2022_2797_MOESM1_ESM.docx]

**Appendix**

**Appendix A**

***Data Preparation***

Time-series data for affect and cognitive performance were created and a ‘time’ variable was defined. This time variable, ranging from 1 to 63, indicates the unit of the time-series analysis and is equivalent to the assessment day.

Missing values were imputed for each patient separately with the ‘Amelia’ R-package^1^. Amelia performs a multiple imputation while allowing time-lagged effects and time trends to be included in the estimation of the missing values. The proportion of missing values ranged from 1% to 26% for negative- and positive affect, and from 3% to 33% for working memory and visual learning. We assume Missing At Random, as the reasons for missings were often technical difficulties (e.g., laptop not working).

We standardized the negative affect, positive affect, working memory and visual learning measures for each person separately as we expected fluctuations (both the amount and magnitude) to be different per person. Each of these variables were divided by the person-specific standard deviation (SD), so that one unit in the variable was equivalent to one person-specific SD, which allowed for easier interpretation.

For some patients the autovar package did not yield valid models because the normality assumption was violated. Therefore, we transformed some of the endogenous variables of these patients before entering them into the autovar model selection procedure.

Two patients started and stopped use of their medication throughout the study period. Three patients changed the dosage of their medication. One patient was also admitted to the psychiatric ward for 38 days.

**Appendix B**

***Description of the ‘autovar’ Modelling Procedure***

To give a more detailed description of the ‘autovar’ modelling procedure, we will describe how valid models were determined. Four assumptions were tested using the ‘autovar’ R-package. First, the stationarity assumption was tested using the Philips Perron test and the eigenvalue test. The eigenvalue test assessed whether all eigenvalues lie in the unit circle. When the stationarity assumption was not met, a variable indicating a linear or quadratic time trend was added to the model. Second, the “white noise” assumption was tested using the Portmanteau test on the residuals, which assesses whether the residuals are serially uncorrelated. Third, the homoscedasticity assumption was tested with the Portmanteau test on the squares of the residuals. Finally, the Skewness test was used to assess normality of the residuals. If any of the last three assumptions were not met, the package evaluated whether adding a lag-2 autoregressive effect, including dummy variables for outliers, or applying a log transformation on the endogenous variables resulted in valid models. If all these tests were non-significant (*p* ≥ .05), the model was considered valid. After checking the assumptions, redundant models were removed from all resulting valid models, meaning that priority was given to simpler models (e.g. valid models with outliers are redundant to the same valid model without outliers). From the remaining models the model with the best fit statistic (AIC/BIC) was chosen as the final model.

**Appendix C**

*Order 2 Cumulative Orthogonalized Impulse Response Function Analysis of Working Memory and Negative Affect for Patient 4*


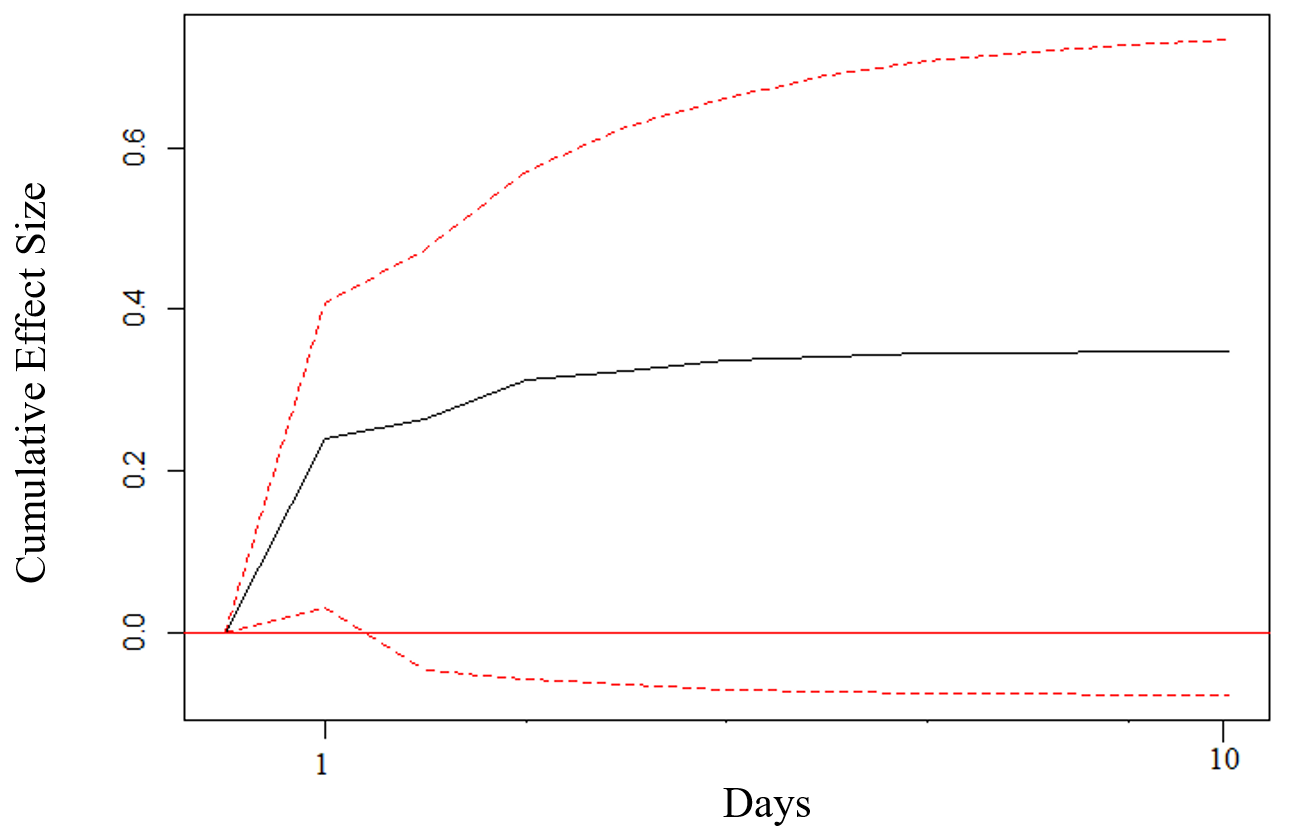


*Note.* Impulse = negative affect. Response = working memory. Order 2 assumes a contemporaneous association in the direction of changes in working memory preceding changes in negative affect. Dotted lines represent the 99% confidence intervals around the cumulative effect sizes.

**Appendix D**

***Information on variability of affect and cognitive performance measures***

Time series plots of each item separately showed that variability of affect items differed per item and per person. There was no item that consistently showed little fluctuations in all persons (see graphs below that show fluctuations for the individual items over time (each graph comprises one participant, the lines comprise the items)). In addition, we have checked the variability of all total scores for cognitive performance and affect for each participant. We defined sufficient variability if: 1) for a specific person <80% of the scores over time were the same^2^, and 2) if the intra-individual variability exceeded 10% of the known between-subject variability^2^. For this latter purpose, we calculated the root-square of the mean squared successive differences (rMSSD)^3^ for these variables for each participant. All total scores for affect and cognitive performance showed sufficient variability in all participants according to these two parameters.

*Variability of affect items for Patient 4*

**
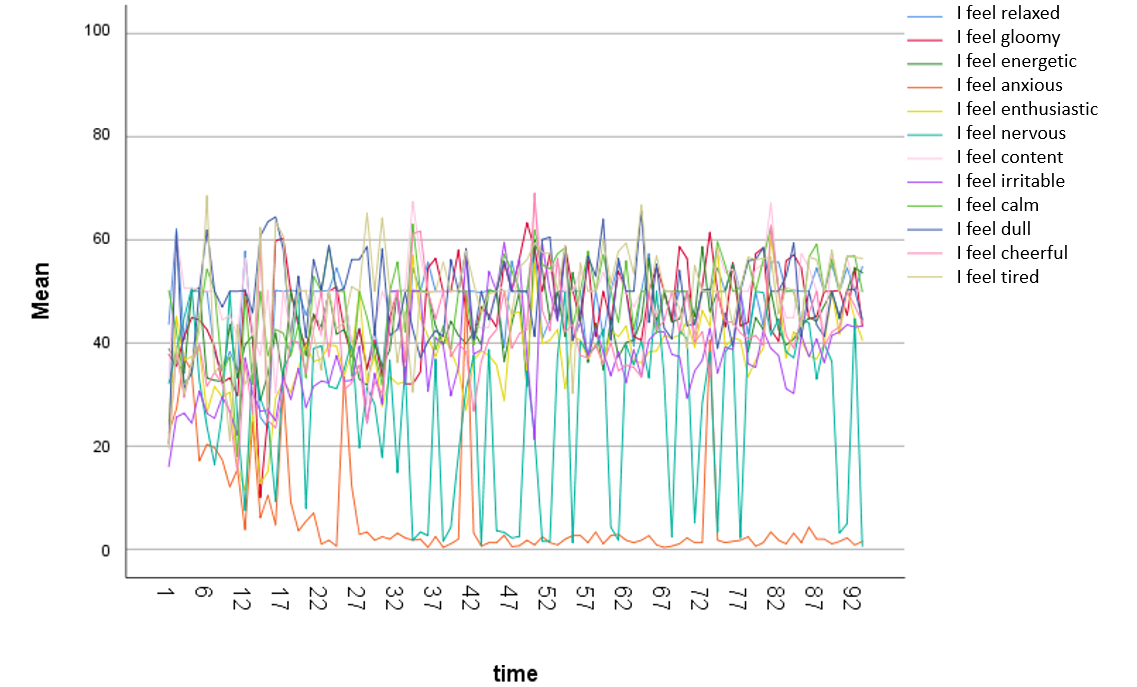
**

*Variability of affect items for Patient 5*

*
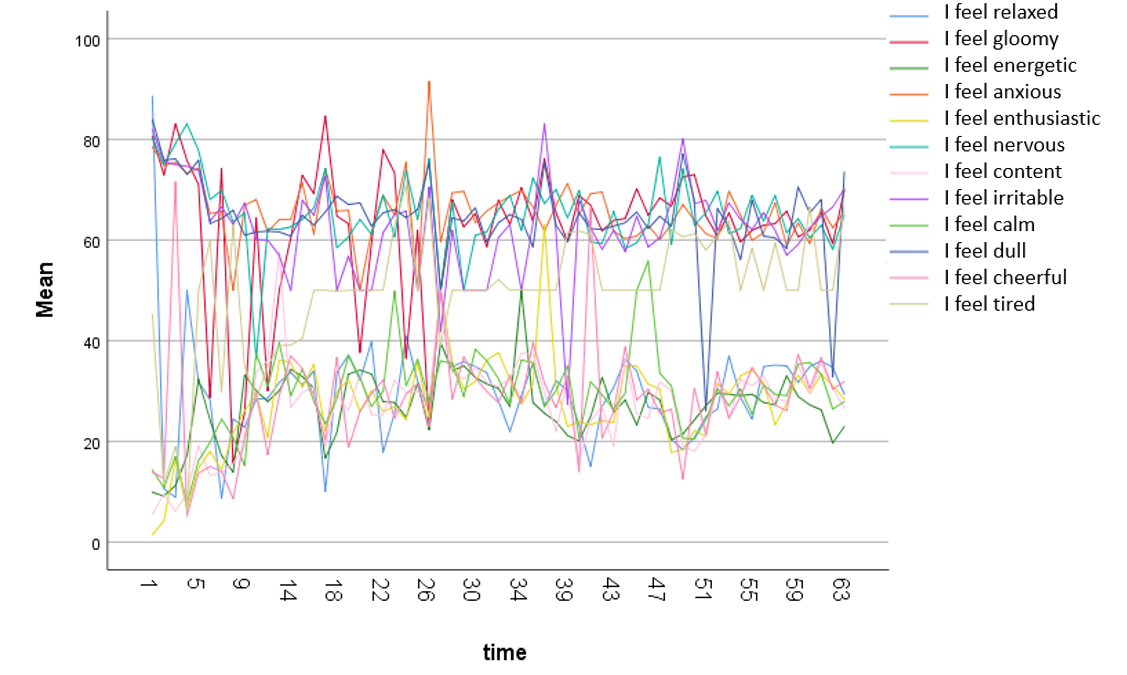
*

*Variability of affect items for Patient 7*


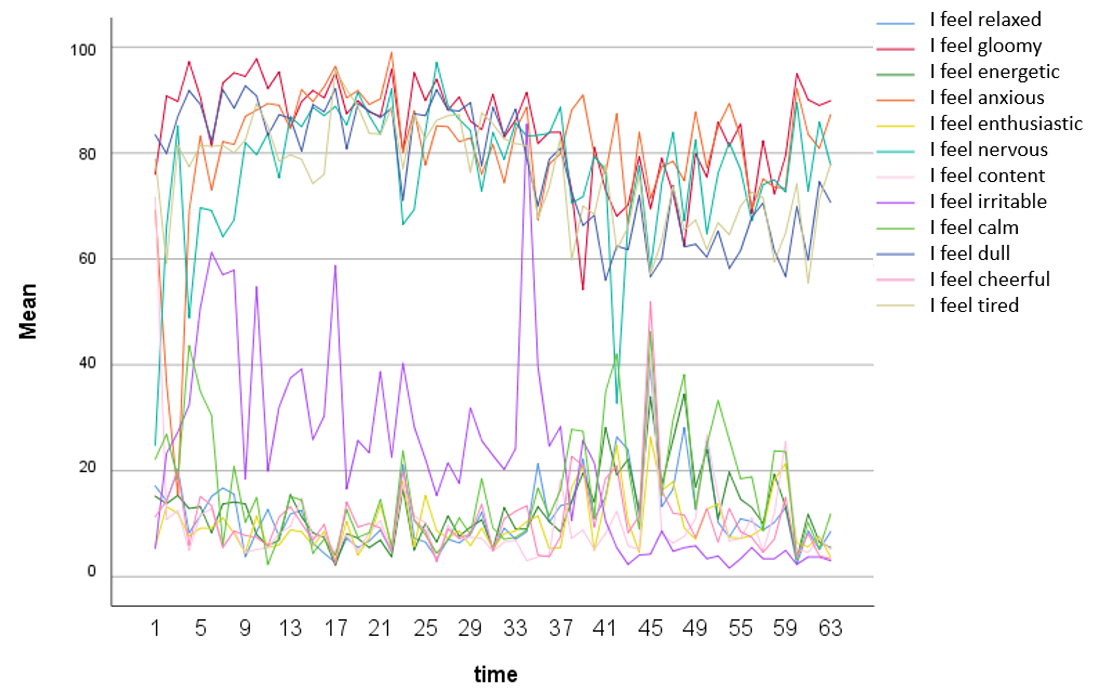


*Variability of affect items for Patient 8*

*
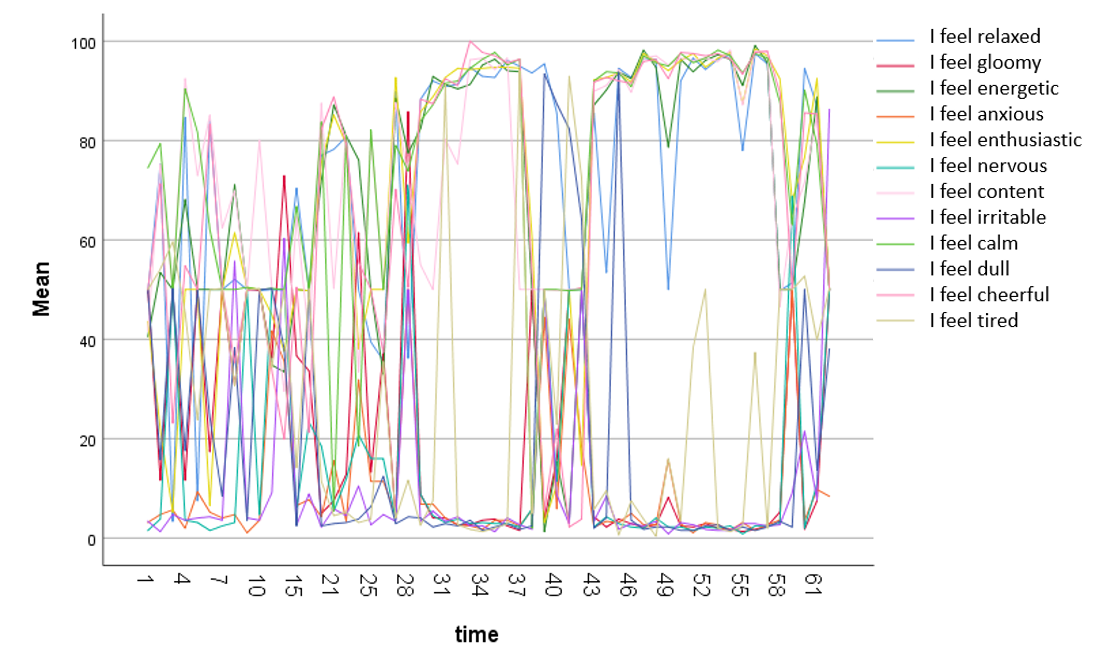
*

*Variability of affect items for Patient 9*

*
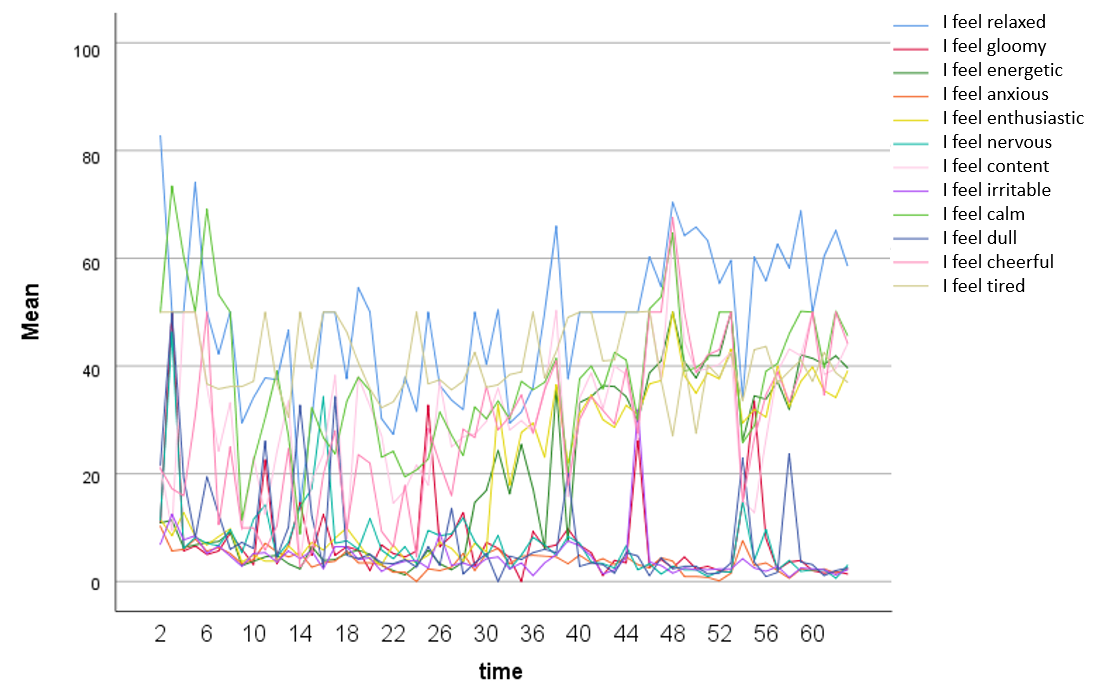
*

*Variability of affect items for Patient 10*

*
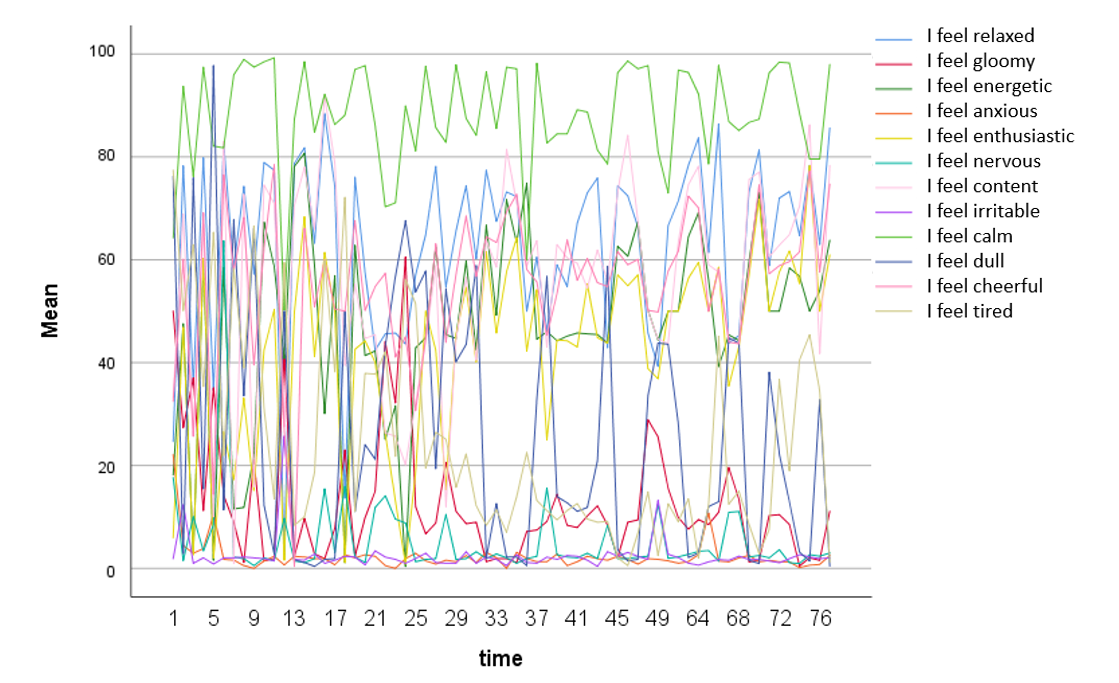
*

*Variability of affect items for Patient 11*

*
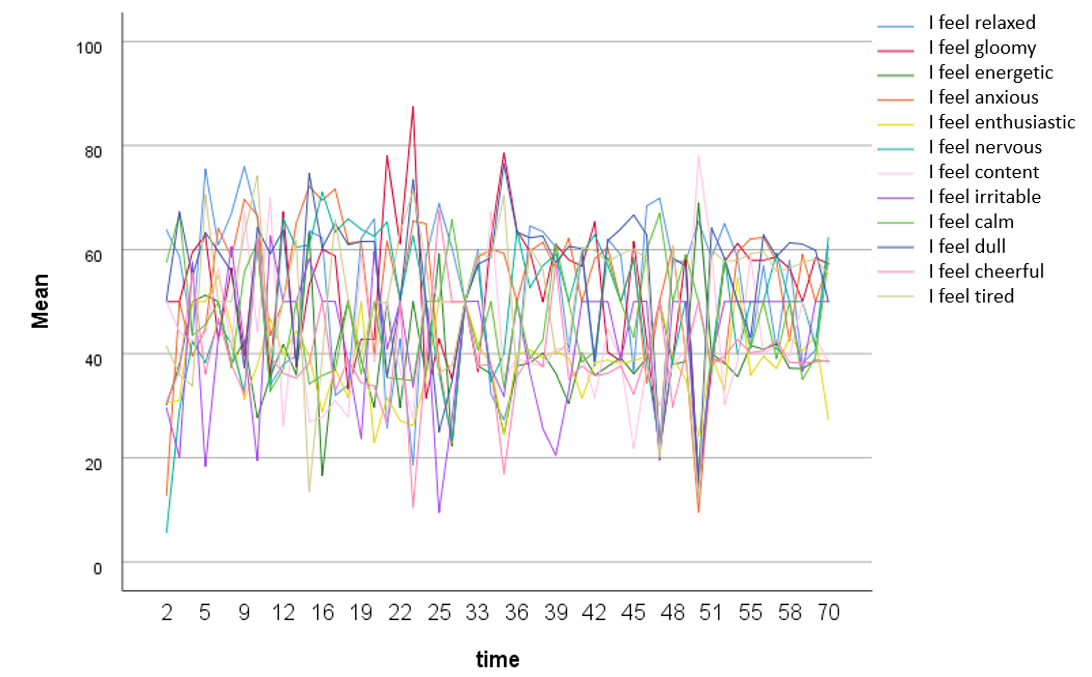
*

**References**

1. Honaker J, King G, Blackwell, M. Amelia II: A program for missing data. Journal of statistical software. 2011;45(7):1-47.
2. Brose A, Ram N. Within-person factor analysis: modeling how the individual fluctuates and changes across time. In: Mehl MR & Conner TS, editors. Handbook of research methods for studying daily life. New York: Guildford Press; 2021. 459-478.
3. von Neumann J, Kent RH, Bellinson HR, Hart BI. The Mean Square Successive Difference. Ann Math Stat. 1941;12(2):153-162. doi:10.1214/aoms/1177731746
